# Supplementary material for: Characterization and Functional Analysis of OcomOBP7 in Ophraella communa Lesage
Source: Insects. 2023 Feb 14;14(2):190. doi: 10.3390/insects14020190 (PMC9967674; doi:10.3390/insects14020190)
Supplement: Supplementary file 1 [file insects-14-00190-s001.zip › insects-2163155-supplementary.pdf]

Supplementary Materials:

Table S1: Sequence BLASTX information for *OcomOBP7* in *O. communa.*

Gene Name	Species Name	Acc. number	Identity (%)
CforOBP26	*Cylas formicarius*	UNA06110.1	44.37
CbowOBP1	*Colaphellus bowringi*	ALR72489.1	54.23
MaltOBP4	*Monochamus alternatus*	AHA39269.1	50.00
BhorOBP1	*Batocera horsfieldi*	AHA33382.1	52.82
XquaOBP8	*Xylotrechus quadripes*	AXO78386.1	48.95
AglaOBP14	*Anoplophora glabripennis*	ARH65469.1	48.59
HaxyOBP7	*Harmonia axyridis*	AVH84914.1	43.66

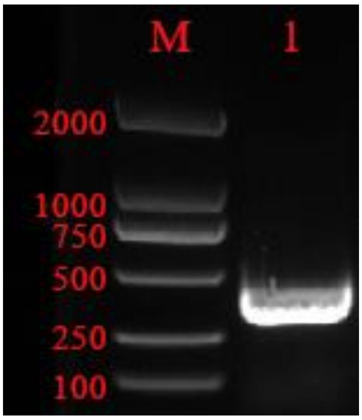

Figure S1. PCR product of *OcomOBP7*. Line M: Trans2K DNA Maker; Line 1: PCR product of *OcomOBBP7* sequence.

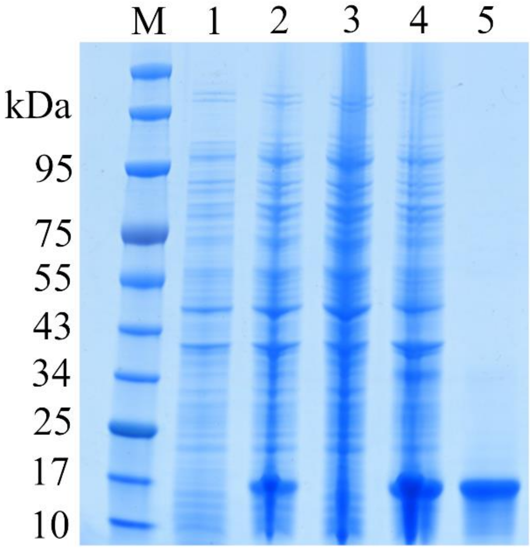

Figure S2. SDS-PAGE of expression and purification of *OcomOBP7*. M: molecular weight marker; 1: noninduced pET28a-OcomOBP7; 2: induced pET28a-OcomOBP7; 3: supernatant of

induced pET28a-OcomOBP7; 4: inclusion body of induced pET28a-OcomOBP7; 5: Purified pET28a-OcomOBP7.

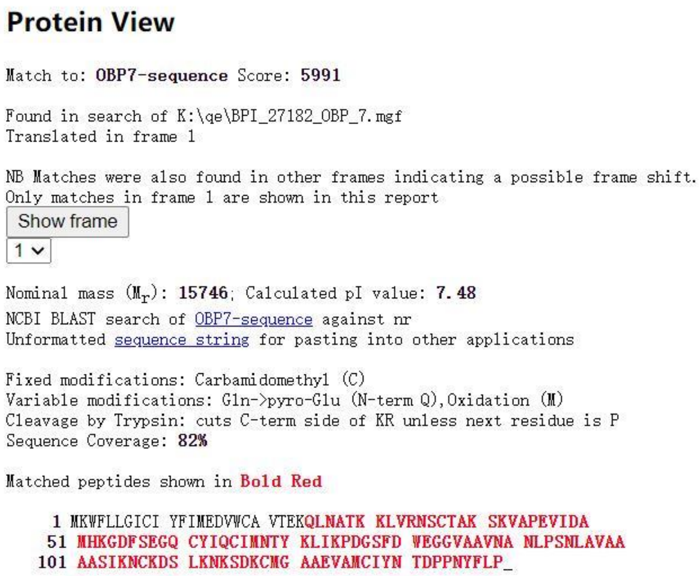

Protein View

Match to: **OBP7-sequence** Score: **5991**

Found in search of K:\qe\BPI_27182_OBP_7.mgf
Translated in frame 1

NB Matches were also found in other frames indicating a possible frame shift.
Only matches in frame 1 are shown in this report

Show frame

1

Nominal mass (M_r): **15746**; Calculated pI value: **7.48**
NCBI BLAST search of OBP7-sequence against nr
Unformatted sequence string for pasting into other applications

Fixed modifications: Carbamidomethyl (C)
Variable modifications: Gln->pyro-Glu (N-term Q),Oxidation (M)
Cleavage by Trypsin: cuts C-term side of KR unless next residue is P
Sequence Coverage: **82%**

Matched peptides shown in **Bold Red**

```
  1 MKWFLLGICI YFIMEDVWCA VTEKQLNATK KLVRNSCTAK SKVAPEVIDA
 51 MHKGDFSEGQ CYIQCIMNTY KLIKPDGSFD WEGGVAAVNA NLPSNLAVAA
101 AASIKNCKDS LKNKSDKCMG AAEVAMCIYN TDPPNYFLP_
```

Figure S3. Results of mass spectrometry identification of OcomOBP7.
